# Supplementary material for: Changes in Mental Health and Preventive Behaviors before and after COVID-19 Vaccination: A Propensity Score Matching (PSM) Study
Source: Vaccines (Basel). 2021 Sep 19;9(9):1044. doi: 10.3390/vaccines9091044 (PMC8473427; doi:10.3390/vaccines9091044)
Supplement: Supplementary file 1 [file vaccines-09-01044-s001.zip › vaccines-1366348-supplementary.pdf]

Supplementary Information

Supplementary Table S1. The reliability and confirmatory analysis of corresponding scales were evaluated according to Cronbach's α coefficient

Variable	Cronbach's α	χ^2/df	RMSEA	CFI	TLI
HBM	0.794	16.758	0.062	0.955	0.911
Mental health	0.813	42.389	0.101	0.944	0.922
Preventive behavior	0.835	31.277	0.086	0.949	0.929
Suggested value for good fit	>0.700	2~5	<0.060	>0.900	>0.900

Footnote: As shown, the Cronbach's α coefficient was used to report the reliability of the scales. The confirmatory analysis (CFA) indexes selected to report the model fit were: χ^2/df, Wheaton et al.'s relative/normed chi-square; RMSEA, the root mean square error approximation; CFI, the comparative fit index; TLI, the Tucker-Lewis index. The following cutoff criteria were used: (a) Cronbach's α of 0.700 or greater; (b) χ^2/df between 2 and 5; (c) RMSEA of 0.060 or less; (d) CFI of 0.900 or greater; and (e) TLI of 0.900 or greater.

Supplementary Table S2. Factors associated with the score of scales by multivariate linear regression.

Variable	HBM scale			Mental health			Preventive behavior		
	B	*β*	*P*	*B*	*β*	*P*	*B*	*β*	*P*
Gender									
Male	ref	ref	ref	NA	NA	NA	ref	ref	ref
Female	0.696	0.047	0.003	NA	NA	NA	0.498	0.047	0.002
Age	NA	NA	NA	0.033	0.062	<0.001	0.023	0.047	0.005
Education									
Junior high school and below	ref	ref	ref	ref	ref	ref	ref	ref	ref
High school degree	1.806	0.103	<0.001	0.022	0.002	0.937	0.173	0.014	0.519
Bachelor's degree	1.81	0.121	<0.001	-1.004	-0.09	<0.001	-0.867	-0.082	0.001
Master's degree or above	1.593	0.05	0.008	-1.339	-0.056	0.003	-1.069	-0.048	0.013
Family per capita monthly income									
<5000	ref	ref	ref	ref	ref	ref	ref	ref	ref
5000-10000	1.459	0.095	<0.001	-0.213	-0.019	0.302	-0.099	-0.009	0.611
10001-15000	1.509	0.067	<0.001	-0.561	-0.033	0.062	-0.154	-0.01	0.588
>15000	2.233	0.096	<0.001	-1.11	-0.064	<0.001	-0.388	-0.024	0.199
Vaccination									
Pre-vaccination	NA	NA	NA	ref	ref	ref	ref	ref	ref
Post-vaccination	NA	NA	NA	-0.616	-0.055	<0.001	0.617	0.059	<0.001

Footnote: B, not standardized regression coefficient; β, standardized regression coefficient; NA, not available.

Supplementary Materials --- Questionnaire used in the survey.

Section A: Demographics

We will now ask you some questions about yourself.

A1. What is your gender?

a. Male

b. Female

A2. What is your age?

(Numeric values from 18 to 80)

A3. What is the highest level of education you have completed? If currently enrolled, mark the highest qualification received.

a. Junior high school and below

b. High school degree

c. Bachelor’s degree

d. Master’s degree or above

A4. What is your family per capita income?

a. <5000 RMB

b. 5000-10000 RMB

c. 10001-15000 RMB

d. >15000 RMB

A5. What is your current state of health?

a. Very good

b. Good

c. General

d. Poor

e. Very poor

A6. Have you taken a flu vaccine in the last three years?

a. No vaccination

b. Irregular vaccination

c. Regular vaccination

A7. In the past year, how often did you take domestic business trip in China?

a. At least twice a month

b. About once a month

c. About once every 3 months

d. About once every 6 months

e. Barely

A8. In the past year, how often did you take foreign business trip out of China?

a. At least once every 3 months

b. About once every 6 months

c. About once a year

d. Barely

A9. When did you get the first shot of the COVID-19 vaccine?

a. Just vaccinated

b. More than 2 weeks

c. Within 1 month

d. Within 3 months

e. Within 6 months

Section B: Health belief model

We will now ask you some questions about COVID-19 and vaccines in general. How strongly do you agree or disagree with each of the following statements?

Rotate Statement	Strongly disagree	Tend to disagree	Do not know	Tend to agree	Strongly agree
B1) I think I have a high probability of infecting COVID-19 in the next 12 months	1	2	3	4	5
B2) I think the children have a high probability of infecting COVID-19 in the next 12 months	1	2	3	4	5
B3) I think the elderly have a high probability of infecting COVID-19 in the next 12 months	1	2	3	4	5
B4) I can't live a normal life like I did before COVID-19 pandemic	1	2	3	4	5
B5) If I am infected with COVID-19, I will think the consequences will be very	1	2	3	4	5

serious					
B6) If I am infected with COVID-19, I think it is very likely to have long-term sequelae	1	2	3	4	5
B7) If the child in the family is infected with COVID-19, I think the consequences will be very serious	1	2	3	4	5
B8) If the elderly in the family is infected with COVID-19, I think the consequences will be very serious	1	2	3	4	5
B9) The novel coronavirus (COVID-19) has a strong ability to spread	1	2	3	4	5
B10) A novel coronavirus vaccination can prevent COVID-19	1	2	3	4	5
B11) A coronavirus vaccine can reduce the severity of complications when people are infected with COVID-19.	1	2	3	4	5
B12) Getting the coronavirus vaccine can prevent people from spreading the COVID-19 to others	1	2	3	4	5
B13) Overall, it is difficult to take COVID-19 vaccine at a community health	5	4	3	2	1

center or a designated vaccination site					
B14) Overall, I think getting coronavirus vaccine will cause a healthy person to be infected with COVID-19	5	4	3	2	1
B15) Overall, I think there may be strong side effects after the COVID-19 vaccine	5	4	3	2	1
B16) I was vaccinated against COVID-19 on the recommendation of the National Department of Disease Control and Prevention	1	2	3	4	5
B17) I was vaccinated against COVID-19 according to the advice of people around me (family, friends, colleagues).	1	2	3	4	5
B18) I was vaccinated against COVID-19 according to the advice of doctors, community workers and other professionals.	1	2	3	4	5

Section C: Questions about your recent mental health status.

How strongly do you agree or disagree with each of the following statements?

Rotate Statement	Strongly disagree	Tend to disagree	Do not know	Tend to agree	Strongly agree

C1) I don't think the COVID-19 pandemic will end soon	1	2	3	4	5
C2) In the past month, my mental state has been negatively affected by the new coronary pneumonia	1	2	3	4	5
C3) In the past month, I have reduced my communication with my family or friends	1	2	3	4	5
C4) In the past month, my sleep quality has deteriorated	1	2	3	4	5
C5) In the past month, I often feel depressed	1	2	3	4	5
C6) In the past month, I often have difficulty concentrating	1	2	3	4	5
C7) In the past month, I often feel irritable	1	2	3	4	5
C8) I think going out to large gatherings puts everyone in danger	1	2	3	4	5
C9) I feel very nervous when I hear people around me coughing or sneezing	1	2	3	4	5

Section D: Questions about your recent preventive behavior performance against COVID-19.

How strongly do you agree or disagree with each of the following statements?

Rotate Statement	Strongly disagree	Tend to disagree	Do not know	Tend to agree	Strongly agree

D1) I will wear a mask as much as possible when I go out	1	2	3	4	5
D2) I will consciously maintain a certain social distance from others	1	2	3	4	5
D3) When going out, I don’t touch my eyes, nose and mouth directly with my hands	1	2	3	4	5
D4) I will wash my hands with soapy water or a disposable sanitizer when I touch something outside	1	2	3	4	5
D5) I try not to touch my family or other things before washing my hands with soap and so on when I go home	1	2	3	4	5
D6) I try to reduce the use of public transport	1	2	3	4	5
D7) I reduced my daily social activities.	1	2	3	4	5
D8) I reduced eating out	1	2	3	4	5
D9) When shopping I prefer online shopping, instead of going to crowded places like supermarkets	1	2	3	4	5
